# Genomic and human papillomavirus profiling of an oral cancer cohort identifies TP53 as a predictor of overall survival

**DOI:** 10.1186/s41199-019-0045-0

**Published:** 2019-12-05

**Authors:** Neil Mundi, Stephenie D. Prokopec, Farhad Ghasemi, Andrew Warner, Krupal Patel, Danielle MacNeil, Christopher Howlett, William Stecho, Paul Plantinga, Nicole Pinto, Kara M. Ruicci, Mohammed Imran Khan, Myung Woul Han, John Yoo, Kevin Fung, Axel Sahovaler, David A. Palma, Eric Winquist, Joe S. Mymryk, John W. Barrett, Paul C. Boutros, Anthony C. Nichols

**Affiliations:** 1 Department of Otolaryngology – Head and Neck Surgery, Western University, London, ON Canada; 2 Victoria Hospital, London Health Science Centre, Room B3-431A, 800 Commissioners Road East, London, ON N6A 5W9 Canada; 3 Ontario Institute for Cancer Research, Toronto, ON Canada; 4 Department of Oncology, Western University, London, ON Canada; 5 Department of Pathology, Western University, London, ON Canada; 6 Department of Otolaryngology, College of Medicine, Ulsan University Hospital, University of Ulsan, Ulsan, South Korea; 7 Department of Microbiology & Immunology, Western University, London, ON Canada; 8 Department of Medical Biophysics, University of Toronto, Toronto, ON Canada; 9 Department of Pharmacology & Toxicology, University of Toronto, Toronto, ON Canada

**Keywords:** Oral cancer, Genomics, Mutational status, tp53, Survival

## Abstract

**Background:**

The genomic landscape of head and neck cancer has been reported through The Cancer Genome Atlas project. We attempt to determine if high-risk human papillomavirus (HPV) or frequently mutated genes are correlated with survival in an oral cancer cohort.

**Methods:**

Patient demographic data along with data from final pathology was collected. Tumor DNA was analyzed using a custom Illumina targeted sequencing panel. Five high-risk HPV types were tested by qPCR. Statistical analyses were used to identify associations between patient outcome and mutational status.

**Results:**

High-risk HPV types were identified in 7% of cases; HPV status was not associated with survival. Mutations were identified in TP53, TERT promoter, & PIK3CA. Mutations in TP53 were significantly associated with poorer overall survival on multi-variate analysis (*p* = 0.03).

**Conclusions:**

Mutations in TP53 were associated with poor patient survival. Expanding our sample size may identify further predictors of outcome to direct customized cancer care.

## Introduction

Oral cavity squamous cell carcinoma (OSCC) is a significant public health problem worldwide, with over 350,000 new cases diagnosed yearly and greater than 150,000 annual deaths [1]. Unfortunately, both the disease process and treatment modalities can have a profound negative impact on patient quality of life, due to difficulty in eating and speaking, disfigurement and chronic pain. In addition, survival rates for OSCC continue to be poor, with fewer than 50% of patients with advanced disease living 5 years following diagnosis [2].

Exposure to tobacco and alcohol represent conventional risk factors for the development of OSCC. However, oral human papillomavirus (HPV) infection has recently been discovered to be an additional risk factor for a significant number of head and neck cancers, particularly those arising in the oropharynx. The most common subtypes identified by The Cancer Genome Atlas (TCGA) were HPV16 (84%), HPV33 (11%), HPV35 (4%) and HPV56 (1%) [3]. Importantly, these HPV related oropharyngeal cancers experience markedly improved survival relative to HPV negative tumors [4–6]. HPV related cancers contribute to a small percentage of OSCC [7], however the prognostic importance of HPV infection in this site remains unclear [8–10].

The large sample size (515 patients) and the robust, high quality tumor molecular data from TCGA head and neck cancer cohort delivers a rich opportunity for biomarker discovery. In particular, genetic changes such as *TP53* mutations and 3p arm deletion events were previously found to be predictors of tumor recurrence and overall patient survival within the TCGA cohort [11]. Importantly, validation of these variants in independent cohorts has been performed, but with varying results due to differences in disease site, treatment rendered and base study population [12–15]. Here, we attempt to expand on these findings by evaluating the association of HPV status on patient survival, as well as the impact of mutation status using a list of frequently mutated genes discovered by TCGA, using a prospectively collected cohort of 135 OSCCs.

## Materials and methods

### Patient population

Ethics approval was obtained from the University of Western Ontario Research Ethics Board (REB 16579). One hundred and thirty-six patients with OSCC treated at the London Regional Cancer Program at the London Health Sciences Centre with primary surgery were prospectively consented and enrolled between 2011 and 2015 (note that 1 patient was later excluded due to poor sequencing quality). Patient primary tumors, matched blood and clinical data was collected, including age at diagnosis, use of tobacco and alcohol, 7th edition American Joint Committee on Cancer (AJCC) TNM stage, treatment regimen, and post-treatment follow up information.

### Sample collection and DNA extraction

Fresh tumor was harvested from the center of the ablation specimen after the resection was complete, with care taken not to disturb the surgical margins. Tumor cellularity of > 70% was confirmed by frozen section analysis. Ten mL of whole blood was obtained by venipuncture or arterial line during the anesthetic for the majority of patients (118/136, 87%). The tumor was placed on ice and transported to the research laboratory, where a portion was frozen and another piece underwent immediate DNA extraction using Qiagen kits (Cat #: 80204). DNA was similarly extracted from blood samples, also using Qiagen kits (Cat #: 51104).

### Library generation and targeted sequencing of oral cavity samples

Nine genes were selected for targeted sequencing based on results from the TCGA Head and Neck Squamous Cell Carcinoma (HNSCC) cohort, including *CDKN2A*, *NOTCH1*, *PIK3CA*, *TP53*, *FAT1*, *CASP8*, *COL11A1*, and *HRAS*, as well as the *TERT* promoter region. A custom capture for these nine genes was designed using the Illumina Ampliseq platform. Tumor and matched blood samples (118 patients with matched blood, 22 tumor alone) were processed at the London Regional Genomics Centre on the Illumina MiSeq platform. FASTQ files were downloaded and aligned to GRCh38 using BWA-MEM (v0.7.15). Duplicates were not marked (due to the highly targeted nature of the data). Indel realignment and recalibration was performed using GATK (v3.7.0). A minimum of 80x (tumor) or 50x (normal) coverage across at least 80% of target sequences was obtained in 78% of the samples (201/257; Additional file 1: Figure S1). A single tumor had very poor coverage and was removed from downstream analyses (Additional file 2: Figure S2). Similarly, a single normal sample also had very poor coverage and was removed. The matched tumor sample was subsequently treated as tumor only. For tumors with a matched normal, germline single nucleotide polymorphisms were called using GATKs HaplotypeCaller (v3.7.0) and filtered for quality and read depth. Somatic single nucleotide variants (SNVs) were identified using MuTect (v1.1.7); for tumors without a matched normal sample, a panel of normals (PoN) [generated using 438 normal samples from the TCGA HNSC dataset (BWA v0.7.12 with hs37d5, GATK v3.4.0, MuTect v1.1.6, converted to GRCh38 coordinates using picard v2.7.1)] was used to remove probable germline variants (along with the following filters: SNV passed quality control, had a minimum read depth of 50x and was present in fewer than 4 samples in the PoN). Somatic SNVs were filtered to remove non-functional, off-target (intronic/intergenic) variants prior to downstream analyses. Statistical analyses were performed in the R statistical environment (v3.4.3), with visualizations generated using the BPG (v5.9.8) with the lattice (v0.20–38) and latticeExtra (v0.6–28) packages.

### Determination of HPV status in oral cavity patients

TaqMan primers and probes that were described previously [6, 16, 17] were used to determine the HPV status of the oral cavity tumor samples for HPV types 16, 18, 33, 35 and 56.

### Statistical analyses

Descriptive statistics were generated for baseline patient, tumor and treatment characteristics for all patients that had successfully sequenced samples (*n* = 135). Univariate and multivariate Cox Proportional Hazards Regression was performed to identify significant (*p* < 0.05) predictors of overall survival (OS) and disease-free survival (DFS). Multivariate analysis was performed by first constructing cox models of OS and DFS with all appropriate variables (as identified by univariate analyses), with a backwards stepwise approach to attain the best fitting models of survival. Kaplan-Meier estimates were generated for OS and DFS for all patients. All statistical analyses were performed in SAS (v9.4; SAS institute, Cary NC) using two-sided statistical testing at the 0.05 significance level.

## Results

### Baseline tumor, patient and treatment characteristics

Patient demographics and clinical characteristics are summarized in Table 1. The majority of patients were male, with a mean age at diagnosis of 62.2 years. Patients had a mean smoking history of 26.2 pack years and 40.6% of patients consumed more than twenty-one alcoholic drinks per week. Five percent of patients had positive margins, however an additional 39% of cases had close margins (defined as < 3 mm at our institution). Adjuvant radiotherapy alone was provided to 45% of patients (61/135), while 26% of patients (35/135) received adjuvant chemoradiation (Table 1). The average follow-up for patients was 32 months.

**Table 1:** Patient Demographics

Variable	Value	Number of Patients
HPV	Negative	123
	Positive	12
Gender	Male	97
	Female	38
Age	Mean = 62.3 SD = 11.1	
Smoking	Heavy (> 20 py)	71
	Light (<= 20 py)	26
	Never (0 py)	38
Alcohol Abuse	Yes	54
	No	81
T stage	T0-T2	57
	T3-T4	78
N stage	N0-N2a	89
	N2b-N3	46
Adjuvant Therapy	None	39
	Radiation	61
	Chemoradiation	35

### Mutational landscape and tumor HPV status

**Fig. 1:**
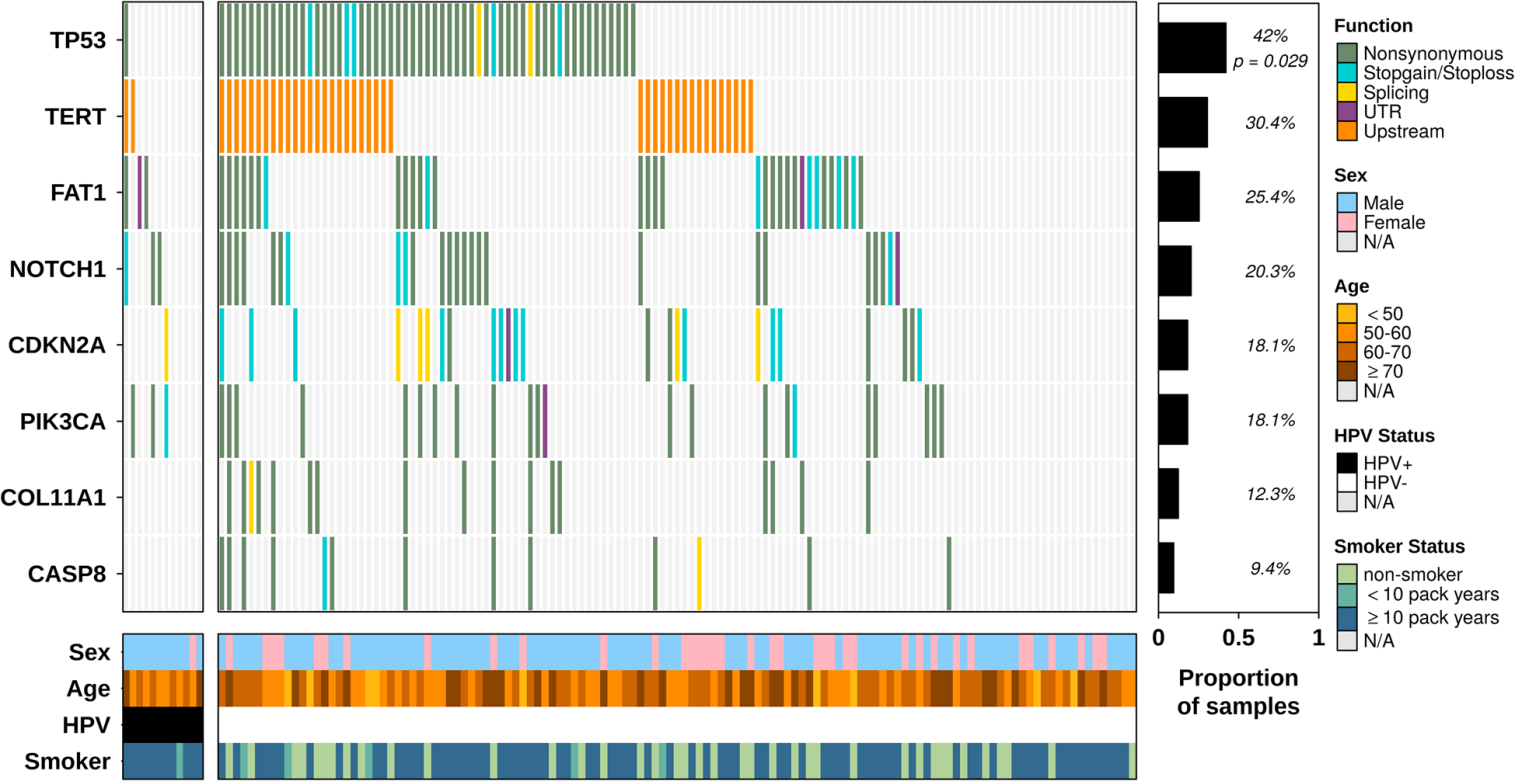
Mutation and HPV status of oral cancer cohort. SNV profile for each gene/patient stratified by HPV-positive and negative cases (on left and right, respectively). Barplot indicates recurrence (proportion of cohort) while covariates show patient sex, age, HPV status and smoking status. *TP53* mutations were enriched in HPV-negative cohort (*p* = 0.029)

Ten tumors (7%) were found to be HPV positive. Of these, nine had evidence of HPV-16 and one carried HPV-33. No tumors were found to harbor HPV types 18, 35 or 56. Gene-wise mutation status for patient tumors and their characteristics are displayed in Fig. 1. A total of 52 unique functional variants were detected in *TP53*, with mutations observed in 42% of patients. Of these, 5 patients each harbored mutations affecting splice sites while 7 harbored nonsense mutations (with 5/7 occurring within exon 6, potentially truncating the DNA-binding domain, Additional file 4: Table S1). The remainder had missense mutations, of which 60% were within the DNA binding domain that are thought to have the greatest impact on function and prognosis [13, 18]. *TP53* mutations were more frequent in HPV-negative tumors (chi-squared proportions test *p* = 0.029). For the remaining targeted genes, *FAT1*, *NOTCH1*, *CDKN2A*, *PIK3CA*, *COL1A11*, and *CASP8,* mutations were identified in 25*,* 20, 18, 18, 12, and 9% of patients respectively (Fig. 1). Unfortunately, the capture for *HRAS* failed for unknown reasons and it was excluded from downstream analyses. The *TERT* promoter was mutated in 30.4% of the samples (Fig. 1), with common variants C228T and C250T observed in 62 and 31% of patients respectively. These appeared to be mutually exclusive. An additional variant, *TERT* C228A, appeared in 3 patients. Clinical variables were assessed for associations with variant status of each gene. *CASP8* and *TERT* promoter mutations were significantly more frequent in patients with less than 10 pack years smoking history (chi-squared proportions test, *p* < 0.01, Additional file 3: Figure S3). *CASP8* mutations were also associated with a younger age at diagnosis (student’s t-test, *p* = 0.009).

### Mutations within TP53 are associated with poor overall survival

**Fig. 2:**
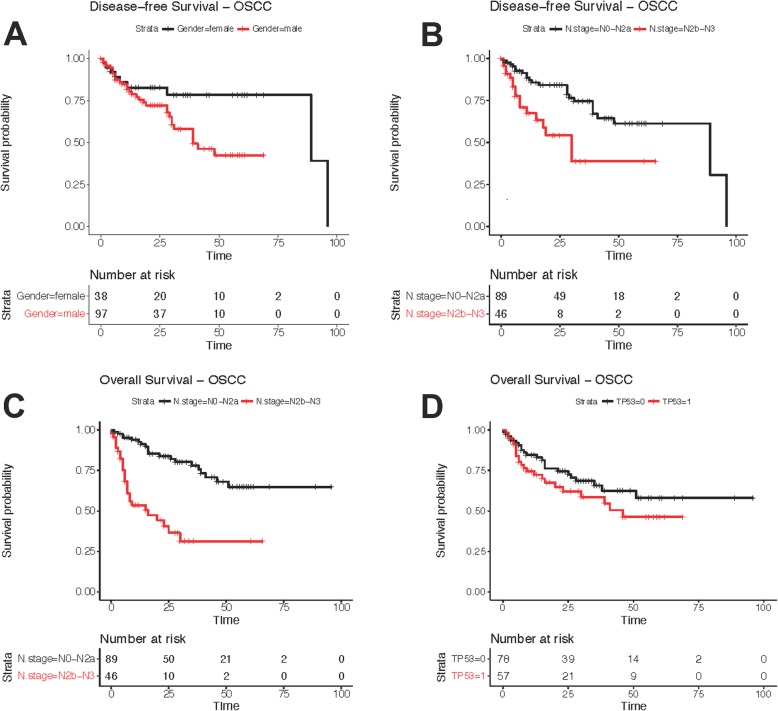
Kaplan-Meier survival curves for clinical factors and genes found to be predictive of overall and disease-free survival through multivariate analysis (Table 2). Age (**a**) and nodal stage (**b**) were predictive of disease-free survival, while advanced nodal stage (**c**) and *TP53* mutation status (**d**) were associated with poorer overall survival

Univariate analysis revealed T and N stage and the use of adjuvant chemotherapy were predictors of poorer overall survival (*p* < 0.05, Additional file 5: Table S2). Female gender, advanced N stage and use of adjuvant chemotherapy were associated with poorer disease-free survival (*p* < 0.05, Additional file 6: Table S3). HPV status and mutations in any gene were not found to be predictive of OS or DFS in univariate analysis (Additional file 5: Table S2 and Additional file 6: Table S3). This was also the case when restricting analysis to mutations within the p53 DNA binding domain. However, known predictors of outcome were significantly associated with OS, including T and N stage (Additional file 5: Table S2 and Additional file 6: Table S3). Backward stepwise multivariate analysis resulted in a model for OS that included age, HPV status, T and N stage and *TP53* mutation status (Table 2). In this model, *TP53* mutations were associated with poorer OS (HR = 1.96 [1.06–3.60], *p* = 0.03; Table 2, Fig. 2).

**Table 2:** Multivariate Analysis of Overall and Disease-Free Survival

Overall Survival				
Variable	Comparison	Hazard Ratio	95% CI	HR *p*-value
Age		1.02	0.993–1.05	0.148
T stage	T3-T4 vs. T0-T2	1.84	0.960–3.53	0.0661
N stage	N2b-N3 vs. N0-N2a	4.63	2.43–8.82	3.16E-06
HPV	Positive vs. negative	2.42	0.817–7.19	0.1107
TP53	Mutant vs. wildtype	1.96	1.06–3.60	0.0311
Disease-Free Survival				
Variable	Comparison	Hazard Ratio	95% CI	HR *p*-value
Gender	Male vs. Female	2.98	1.27–7.01	0.0122
N stage	N2b-N3 vs. N0-N2a	3.12	1.52–6.40	0.00188
HPV	Positive vs Negative	2.25	0.847–5.96	0.104
Adjuvant Chemotherapy	Yes vs. No	1.77	0.885–3.52	0.107

## Discussion

Biomarker studies are frequently hampered by low sample numbers with poor statistical power, the lack of reproducibility of the assay, the heterogeneity of the treatment delivered and the limitations of retrospectively collected data [18, 19]. Here, we performed genomic characterization and HPV typing of a large, prospectively collected cohort of oral cavity cancers treated according to National Comprehensive Cancer Network guidelines, paired with high quality Illumina sequencing, rigorous bioinformatics quality metrics and HPV testing methods that have been thoroughly validated by our group [6, 16, 17, 20]. Importantly, matched normal DNA was available for the majority of cases (87%) to serve as a reference control, which our group has recently demonstrated is critical to generating highly accurate mutation calls (Sun et al., *under revision at Nature Methods*). Thus, with high quality sequencing, matched DNA, prospectively collected tumors and clinical data, we aimed to generate a robust cohort to examine molecular features able to predict patient outcome. We found that the mutational landscape and frequency of HPV-positive disease (7%) was similar to existing OSCC literature [7, 21, 22]. Su et al. demonstrated a mutation frequency of 42.5% in 136 oral cavity squamous cell tumors, while Poeta et al. sequenced 420 patients with HNSCC and found the *TP53* mutation frequency in oral cavity samples to be 53% which are similar to our mutation frequency of 42% [23, 24]. However, the mutation frequency we observed was lower than that of the TCGA analysis and the investigation by Pickering et al. [21, 22]. This heterogeneity of results may be the result of a number of factors including the large number of institutes contributing to the TCGA study versus our biobank collection derived from a single geographic region. In addition, the fact that our analysis is limited to only the oral cavity may also contribute to the differences mentioned. No genes were found to predict survival on their own; however, in multivariate analysis *TP53* mutations were associated with poorer OS, consistent with other reports [11, 13–15, 25].

The p53 protein encoded by *TP53* is a transcription factor, which is often referred to as the “cellular gatekeeper” due to its role in protecting cellular integrity by directing the cell’s physiological response to insults such as DNA damage and hypoxia. *TP53* is the most frequently mutated gene in human cancers (~ 50–60% of cancers) [19]. In head and neck cancer, over 80% of HPV-negative tumors harbor *TP53* mutations, compared to 3% of HPV-positive tumors [21]. In agreement with this, we observed that all but one of the *TP53* mutations occurred in the HPV-negative cohort. It was previously suggested that because the HPV-oncoprotein E6 leads to degradation of p53, selective pressure for loss of function mutations in this gene are unnecessary [26].

Previous analyses of TCGA’s HNSCC cohort identified *TP53* mutations as a predictor of poorer overall survival, with a hazard ratio (HR) of 2.8 ± 0.8 [11]. This was particularly true for missense mutations occurring within the 190 codons of the DNA-binding domain of P53, as well as for splicing and non-sense mutations [8]. However, even mutations that were not anticipated to impact P53 function were found to predict poorer survival compared to wild-type tumors (2.2 ± 0.7). Neskey and colleagues further examined the OSCCs within TCGA that were treated with primary surgery with or without adjuvant therapy (*n* = 168), as well as a separate validation cohort of 96 OSCC patients [13]. They classified tumors with non-synonymous P53 mutations using a novel evolutionary action (EA) score to stratify patients into low- and high-risk groups [13], revealing P53 to be a significant predictor of patient outcome. We have carefully reviewed this data and attempted to classify our observed mutations using the published website for the EA score: http://mammoth.bcm.tmc.edu/EAp53/. We noted that the score cannot be calculated for nonsense and splicing mutations, which make up a significant portion of our *TP53* mutations (12/52, 23%). In addition, the website was not able to calculate a score for some of our missense variants. Thus, we did not include the EA score in our survival analysis. We did not identify any specific mutation groups associated with OS or DFS (Additional file 5: Table S2 and Additional file 6: Table S3). However, P53 mutations, when accounting for other patient and tumor characteristics, were found to be significant for OS (HR = 1.96 [95%: 1.06–3.60]). However, examination of the survival curves in the aforementioned studies, as well as the fact that P53 mutation status did not also predict DFS in our study, begs the question of whether this marker is accurate enough to guide therapeutic decisions. Certainly, P53 mutation status it is not as strong of a predictive marker as p16 in the oropharynx, which has an HR of 0.28 in the context of prospective randomized trials [4]. Likely, P53 mutation status will have to be paired with other markers with molecular and cellular features of tumors that may offer better predictive markers for therapy to build a more reliable predictive model.

In TCGA’s HNSCC cohort, a subset of tumors were enriched for both *CASP8* and *HRAS* mutations [21]. Interestingly, these tumors tend to be P53 wild-type and have a quiescent copy number profile [21]. In vivo, *CASP8*-mutant xenografts have been found to have significantly higher engraftment rates and tumor burden, and lower survival compared to wild-type xenografts [22], suggesting that tumors carrying these mutations may be more aggressive. However, we observed no correlation between *CASP8* mutations and survival, in either TCGA or the current cohort [21]. This may reflect the low frequency of *CASP8* mutations in both datasets, and suggests a much larger sample size may be required to tease out these details.

Tumors carrying activating mutations in the *TERT* promoter region tend to be more aggressive, both in head and neck and other cancer types [12, 27–31]. Evidence exists to suggest that these mutations are overrepresented in patients failing their first line of treatment [12]. Despite being detected in a high fraction of our cohort (30.4%), *TERT* promoter mutations were not associated with OS or DFS. Morris et al. studied a cohort of recurrent and/or metastatic head and neck and cancers and identified *TERT* promoter mutations exclusively in HPV-negative patients (16/29) with none detected in HPV-positive patients (*n* = 20) [12]. This is consistent with the ability of the HPV oncoprotein E6 to activate telomerase independently [32], potentially precluding the need for an activating mutation in the promoter region. We surprisingly identified *TERT* promoter mutations in two HPV-positive patients, which we separately confirmed by conventional and real-time qPCR (data not shown). Further study is needed to understand the role of TERT in both HPV-positive and negative disease.

Despite our concerted efforts to develop a robust biomarker for patient outcome in OSCC, our study faced a number of limitations: the failure of the HRAS capture, lack of matched normal DNA for 22 samples, the small number of genes tested, and a sample size of 135. These limitations will need to be addressed in future studies to identify effective biomarkers of failure that can be translated to clinical care.

The data and conclusions reported herein represent an addition to a growing collection of evidence of the impact of P53 mutations on survival in head and neck cancer. Sandulache et al. recently reported an association between high-risk *TP53* mutations and extra-nodal extension in oral cavity cancer for example [15]. Lapke et al. observed that missense mutations in the *TP53* DNA-binding domain were an independent prognostic factor for shorter disease-free survival [33]. Investigations such as these and our own serve to further validate the findings of the TCGA. In our case, we studied data obtained from patients treated at a single surgical center with a single disease subsite using a streamlined treatment protocol; importantly, this served to control for confounding factors that can influence the fidelity of patient outcome data.

## Conclusions

The mutational landscape and frequency of HPV detection in OSCC was consistent with the literature. Mutations in *TP53* were associated with poor overall survival, highlighting this gene as a potential biomarker for prediction of patient outcome. Expanding our sample size and increasing the number of genetic features studied may identify further predictors of outcome to direct customized patient care.

## Supplementary information

**Additional file 1: Figure S1.** Following alignment, coverage of target sequences was evaluated for each sample. A minimum of 80x (tumor) or 50x (normal) coverage across at least 80% of target sequences was obtained in 78% of samples (201/257).

**Additional file 2: Figure S2.** A single tumor sample [504] had very poor coverage and was removed from downstream analyses. Similarly, a single normal sample [395] also had very poor coverage and was removed. The matched tumor sample was subsequently treated as tumor only.

**Additional file 3: Figure S3.** Comparison of mutations by smoking status. *CASP8* and *TERT* promoter mutations occurred more frequently in patients with less than 10 pack year smoking history.

**Additional file 4: Table S1.** Summary of *TP53* mutations.

**Additional file 5: Table S2.** Univariate Analysis for Overall Survival.

**Additional file 6: Table S3.** Univariate Analysis for Disease-free Survival.

## Data Availability

Please contact author for data requests.

## Abbreviations

AJCC: American Joint Committee on Cancer
DFS: Disease-Free Survival
HNSCC: Head and Neck Squamous Cell Carcinoma
HPV: Human Papilloma Virus
OS: Overall Survival
OSCC: Oral Squamous Cell Carcinoma
SNV: Single Nucleotide Variation
TCGA: The Cancer Genome Atlas
