# Supplementary figures and images for: Genomic and human papillomavirus profiling of an oral cancer cohort identifies TP53 as a predictor of overall survival
Source: Cancers Head Neck. 2019 Dec 5;4:5. doi: 10.1186/s41199-019-0045-0 (PMC6894507; doi:10.1186/s41199-019-0045-0)

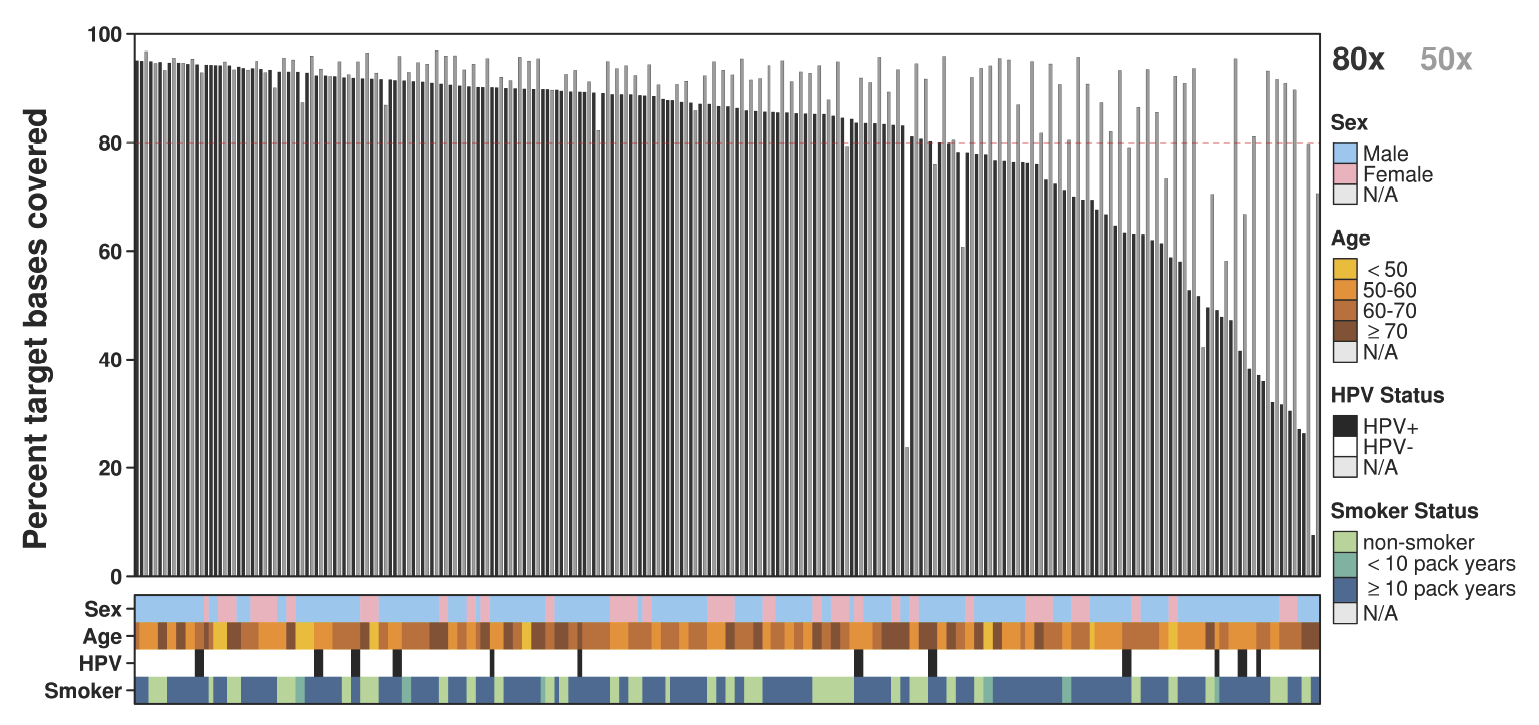

Supplement: Supplementary file 1 — Additional file 1: Figure S1. Following alignment, coverage of target sequences was evaluated for each sample. A minimum of 80x (tumor) or 50x (normal) coverage across at least 80% of target sequences was obtained in 78% of samples (201/257). [file 41199_2019_45_MOESM1_ESM.tiff]

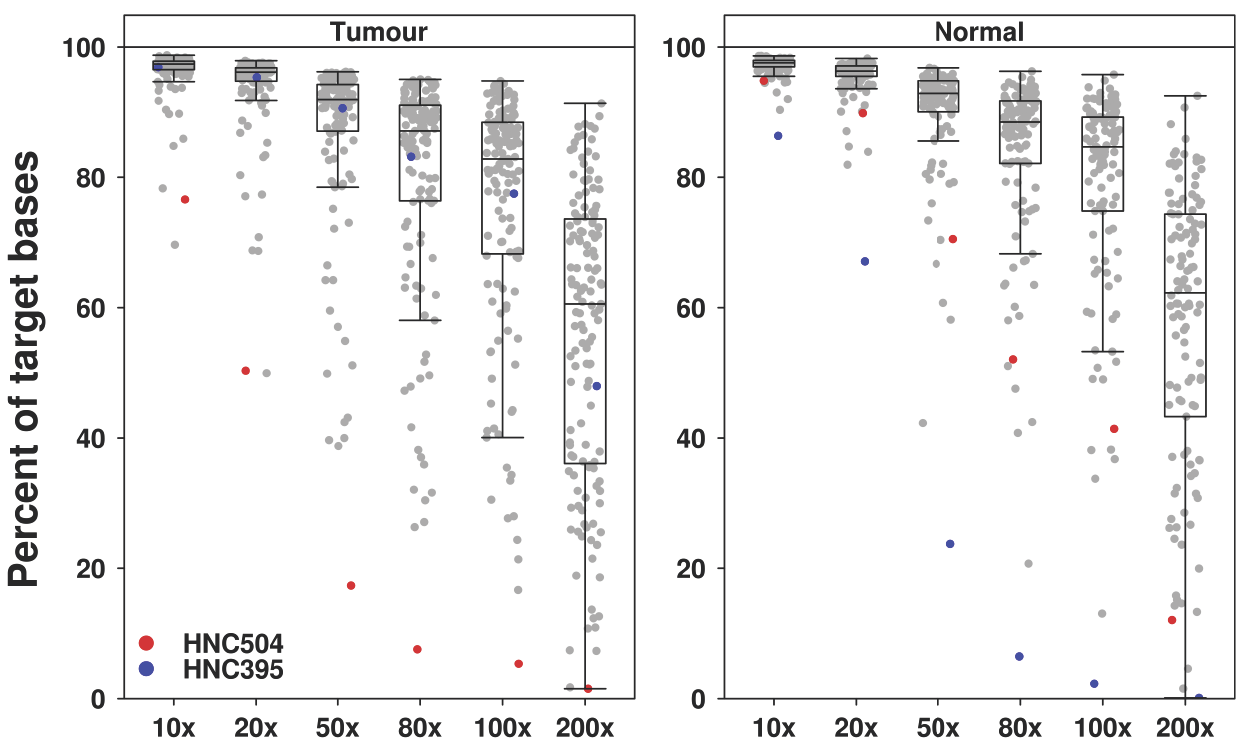

Supplement: Supplementary file 2 — Additional file 2: Figure S2. A single tumor sample [504] had very poor coverage and was removed from downstream analyses. Similarly, a single normal sample [395] also had very poor coverage and was removed. The matched tumor sample was subsequently treated as tumor only. [file 41199_2019_45_MOESM2_ESM.tiff]
