# Supplementary material for: Genomic and human papillomavirus profiling of an oral cancer cohort identifies TP53 as a predictor of overall survival
Source: Cancers Head Neck. 2019 Dec 5;4:5. doi: 10.1186/s41199-019-0045-0 (PMC6894507; doi:10.1186/s41199-019-0045-0)
Supplement: Supplementary file 4 — Additional file 4: Table S1. Summary of TP53 mutations. [file 41199_2019_45_MOESM4_ESM.docx]

Supplementary Table 1. Summary of *TP53* mutations

| Splicing Mutations | | | | | |
| --- | --- | --- | --- | --- | --- |
| Chrom | Position | Ref | Alt | Location | COSMIC.ID |
| chr17 | 7669692 | T | C | intron10 | COSM1191161 |
| chr17 | 7674179 | A | C | intron7 | COSM3728361 |
| chr17 | 7674180 | C | T | intron7 | COSM43571 |
| chr17 | 7674858 | C | T | intron6 | COSM6906 |
| chr17 | 7675052 | C | G | intron5 | COSM33648 |
| Missense Mutations | | | | | |
| Chrom | Position | Ref | Alt | Location | COSMIC.ID |
| chr17 | 7673704 | G | A | exon8 | COSM10663 |
| chr17 | 7674894 | G | A | exon6 | COSM10654 |
| chr17 | 7674945 | G | A | exon6 | COSM10705 |
| chr17 | 7674957 | G | A | exon6 | COSM10733 |
| chr17 | 7676211 | C | T | exon4 | COSM44760 |
