# Supplementary material for: Genomic and human papillomavirus profiling of an oral cancer cohort identifies TP53 as a predictor of overall survival
Source: Cancers Head Neck. 2019 Dec 5;4:5. doi: 10.1186/s41199-019-0045-0 (PMC6894507; doi:10.1186/s41199-019-0045-0)
Supplement: Supplementary file 5 — Additional file 5: Table S2. Univariate Analysis for Overall Survival. [file 41199_2019_45_MOESM5_ESM.docx]

Supplementary Table 2. Univariate Analysis for Overall Survival

| Variable | Comparison | Hazard Ratio | 95% CI | HR p-value |
| --- | --- | --- | --- | --- |
| Gender | Male vs female | 1.358 | 0.702-2.62 | 0.364 |
| Age | | 1.02 | 0.991-1.04 | 0.205 |
| Alcohol abuse | yes vs. no | 1.66 | 0.928-2.96 | 0.0877 |
| Smoking | Light vs. Never | 0.926 | 0.378-2.27 | 0.867 |
|  | Heavy vs. Never | 1.38 | 0.695-2.74 | 0.357 |
| T stage | T3-T4 vs. T0-T2 | 2.17 | 1.16-4.08 | 0.0159 |
| N stage | N2b-N3 vs. N0-N2a | 4.67 | 2.55-8.57 | 6.39E-07 |
| Adjuvant Radiotherapy | Yes vs. No | 1.87 | 0.900-3.87 | 0.0937 |
| Adjuvant Chemotherapy | Yes vs. No | 1.87 | 1.03-3.38 | 0.0387 |
| HPV | Positive vs. negative | 1.12 | 0.400-3.12 | 0.831 |
| TP53 | Mut vs. wildtype | 1.47 | 0.825-2.62 | 0.191 |
| TP53 DNA binding domain | Mut vs. wildtype | 1.42 | 0.796-2.54 | 0.236 |
| PIK3CA | Mut vs. wildtype | 0.865 | 0.403-1.86 | 0.710 |
| CASP8 | Mut vs. wildtype | 0.967 | 0.347-2.71 | 0.954 |
| FAT1 | Mut vs. wildtype | 0.642 | 0.309-1.33 | 0.234 |
| TERT promoter | Mut vs. wildtype | 0.592 | 0.300-1.17 | 0.131 |
| CDKN2A | Mut vs. wildtype | 0.681 | 0.304-1.52 | 0.350 |
| NOTCH1 | Mut vs. wildtype | 1.26 | 0.623-2.55 | 0.519 |
