# Supplementary material for: Genomic and human papillomavirus profiling of an oral cancer cohort identifies TP53 as a predictor of overall survival
Source: Cancers Head Neck. 2019 Dec 5;4:5. doi: 10.1186/s41199-019-0045-0 (PMC6894507; doi:10.1186/s41199-019-0045-0)
Supplement: Supplementary file 6 — Additional file 6: Table S3. Univariate Analysis for Disease-free Survival. [file 41199_2019_45_MOESM6_ESM.docx]

Supplementary Table 3. Univariate Analysis for Disease-free Survival

| Variable | Comparison | Hazard Ratio | 95% CI | HR p-value |
| --- | --- | --- | --- | --- |
| Gender | Male vs female | 2.30 | 1.011-5.22 | 0.047 |
| Age | | 1.00 | 0.976-1.033 | 0.771 |
| Alcohol abuse | yes vs. no | 1.49 | 0.795-2.81 | 0.212 |
| Smoking | Light vs. Never | 1.24 | 0.527-2.93 | 0.620 |
|  | Heavy vs. Never | 1.14 | 0.540-2.43 | 0.724 |
| T stage | T3-T4 vs. T0-T2 | 1.12 | 0.601-2.10 | 0.718 |
| N stage | N2b-N3 vs. N0-N2a | 2.88 | 1.49-5.56 | 0.00158 |
| Adjuvant Radiotherapy | Yes vs. No | 1.80 | 0.825-3.92 | 0.140 |
| Adjuvant Chemotherapy | Yes vs. No | 1.98 | 1.05-3.73 | 0.0359 |
| HPV | Positive vs. negative | 1.83 | 0.716-4.70 | 0.206 |
| TP53 | Mut vs. wildtype | 1.26 | 0.671-2.37 | 0.472 |
| TP53 DNA binding domain | Mut vs. wildtype | 1.32 | 0.701-2.47 | 0.392 |
| PIK3CA | Mut vs. wildtype | 1.19 | 0.565-2.51 | 0.647 |
| CASP8 | Mut vs. wildtype | 1.02 | 0.352-2.93 | 0.978 |
| FAT1 | Mut vs. wildtype | 0.727 | 0.345-1.53 | 0.401 |
| TERT promoter | Mut vs. wildtype | 0.799 | 0.403-1.58 | 0.520 |
| CDKN2A | Mut vs. wildtype | 0.535 | 0.209-1.37 | 0.192 |
| NOTCH1 | Mut vs. wildtype | 1.02 | 0.449-2.33 | 0.959 |
